# 

**Published:** 1875-10

**Authors:** 


### CONTENTS.

PAGE.

**CONTRIBUTIONS.**

The Joys of Misery. Ben. H. Pratt .....58
Wages of Law. P. H. Hayes .....59
American Pharmaceutical Association. Pharmacist. ..60
Op den Graeff .....61

**MEDICAL ITEMS AND NEWS.**

Local Anæsthesia—Rheumatism—To remove India-Ink Marks—Antidote to Carbolic Acid--Method for Administering Raw Meat—Carbuncle—Biliary Calculi—Administration of Chloral—Asthma—Tampon—Acute Mania—Warts—Camphor Ointment—Cystitis—Obstetrical Tincture—Skin-Grafting Superseded—Removal of External Hemorrhoids—Chronic Hydrocephalus—Iodized Glycerine—Milk of Almonds—Diarrhœa Mixture—Cod Liver Oil Palatable--Toothache—Dyspepsia of Smokers--Croton Chloral Hydrate--Antispasmodic Pills—Chloral Suppositories—Peritonitis—Elastic Ligature—Diarrhœa—Plugging Posterior Nares—Antidote to Arsenic—Tincture of Arnica Dangerous—Rheumatism—Diarrhœa of Phthisis—Sore Nipples—Compressed Sponge for Abscess—Croton Chloral in Diseases of Children—Liq. Ferri Perchloridi in Cancer of Uterus—Typhoid Fever—Diarrhœa—Bronchial Catarrh and Winter Cough—Diabetes Millitus—Treatment of Fever—Insulation of Bed in Rheumatism—Biliary Calculi—Hysteria—Delerium Tremens—Dysentery—Antidote to Strychnia—State of Pupil in Chloroform as Indication of Danger—Gastro Enteritis—Tape Worm—Chronic Bronchitis—Examination of Urine .....62 to 72

PAGE.

**OUR CORRESPONDENCE.**

Ten Letters of Interest with Replies .....73

**HOUSEHOLD HINTS AND RECIPES.**

Pretty Red Dye for Wood—Potatoe Sautees—Potatoe Soufflees—Violet Powder—Erasive Soap, to remove grease and stains from clothing—Puree of Potatoes—Highly Illuminating Oil—Harmless Cosmetic Powders—Adhesive Labels—Rhubarb Marmalade .....75

**EDITORIAL.**

Cataract, (illustrated) .....76
Report of Last Fifty Operations for Cataract .....80
Ankle Joint .....80

**EDITORIAL CHIT-CHAT.**

W. F. C.—Photography—New Local—Park Church—The Colored Company—Mailing Machine—Entertaining—Bistoury Wanted—State Fair—Sixteen in One Bed—Vick—Lecture Course—Asked for a Letter—Elmira Driving Park—Advertiser's City Editor—The Vine Stub—Clara Morris and the Moxa—Wrought Iron Furnaces—Reading in Bed—Sectarian Schools—A Puppy—Search in the Dark .....81

